# From Variant of Uncertain Significance to Likely Pathogenic: Adult-Onset Nephronophthisis Linked to NPHP4 p.T680M

**DOI:** 10.1016/j.ekir.2025.09.052

**Published:** 2025-10-14

**Authors:** Neriman Sıla Koç

**Affiliations:** 1 Department of Internal Medicine, Division of Nephrology, Ankara Etlik City Hospital, Ankara, Türkiye

**To the Editor:** König *et al.*^1^ evaluated kidney survival in 383 genetically characterized patients with nephronophthisis (NPHP), highlighting how variant type and clinical features influence renal outcomes. Their study provides important prognostic insights that may guide individualized counseling of patients with NPHP. Here, I describe a consanguineous family with late-onset NPHP4-related disease, thereby extending the clinical spectrum of NPHP4-associated nephronophthisis.

**Figure 1:**
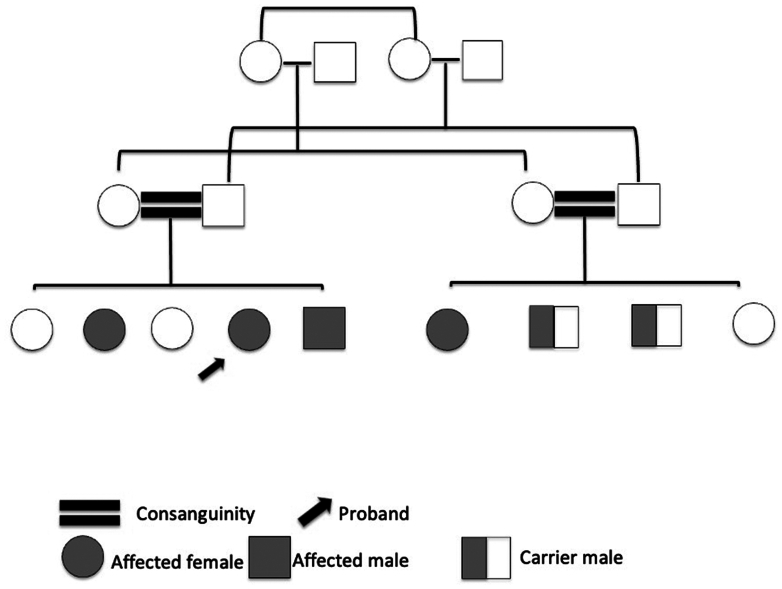
Pedigree of the family with homozygous NPHP4 p.T680M variant. The proband is indicated with an arrow. Shaded circles and squares represent affected females and males, respectively; half-shaded squares indicate heterozygous carrier males; double horizontal lines indicate consanguinity.

The family comprised 4 members with chronic kidney disease manifesting in the third decade of life. Three affected individuals were confirmed to carry the homozygous NPHP4 c.2039C>T (p.T680M) variant, currently listed in ClinVar as of uncertain significance (ClinVar Variation ID: 968861).^2^ Two unaffected brothers were heterozygous carriers, whereas 1 affected sister remains untested. Three individuals progressed to end-stage kidney disease between ages 30 and 41 years, and 1 remains with advanced chronic kidney disease. Ocular findings in this family ranged from absent to severe retinal dystrophy, consistent with the variable ocular expressivity described in NPHP-related ciliopathies.^3^ Kidney biopsy in the proband demonstrated predominant tubulointerstitial scarring, in keeping with NPHP pathology. The pedigree of the family, showing affected and carrier individuals, is presented in Figure 1.

Although NPHP is classically a childhood disease, adult-onset presentations are exceedingly rare, accounting for < 1% of reported cases.^1^ The consistent segregation of the p.T680M variant with disease phenotype in this family suggests pathogenic potential, despite its current classification. Similar to previous reports,^4^^,^^5^ my observations highlight that NPHP4 mutations may underlie adult-onset chronic kidney disease, particularly in consanguineous populations.

In conclusion, this report expands the clinical spectrum of NPHP4-associated nephronophthisis and emphasizes the importance of considering NPHP in adults with unexplained chronic kidney disease. Functional assays and exome-wide studies are warranted to clarify the pathogenicity of the p.T680M variant.
